# Opportunities for Employers to Support Physical Activity Through Policy

**DOI:** 10.5888/pcd16.190075

**Published:** 2019-06-27

**Authors:** Elizabeth Ablah, Stephenie C. Lemon, Nicolaas P. Pronk, Janet R. Wojcik, Qaiser Mukhtar, Jessica Grossmeier, Keshia M. Pollack, Laurie P. Whitsel

**Affiliations:** 1 University of Kansas School of Medicine, Wichita, Wichita, Kansas; 2 University of Massachusetts, Worcester, Massachusetts; 3 HealthPartners Institute and Harvard T.H. Chan School of Public Health, Boston, Massachusetts; 4 Winthrop University, Rock Hill, South Carolina; 5 Centers for Disease Control and Prevention, Atlanta, Georgia; 6 Health Enhancement Research Organization, Waconia, Minnesota; 7 Johns Hopkins Bloomberg School of Public Health, Baltimore, Maryland; 8 American Heart Association, Washington, District of Columbia

In an effort to improve health and business outcomes, workplaces are supplementing traditional physical activity programs focused on individual behavior change with policies designed to change workplace culture. However, confusion exists about how to define workplace policies. In practice, worksites implement programs, benefit designs, and environmental strategies and describe these as policies. The purpose of this essay is to provide a definition for worksite policy and discuss how policy approaches can support employers’ efforts to promote physical activity. We also describe worksite physical activity policies that employers can adopt and implement.

## Defining Worksite Physical Activity Policy

Policies are an increasing focus of workplace wellness initiatives although they are poorly defined. Academic literature includes interventions described as policies that range from resource materials (information) and exercise classes (program) to subsidizing gym memberships (benefit design) and providing accessible stairways (environmental). Developing a standardized definition of worksite physical activity policies may help clarify their effect.

Schmid and colleagues conceptualized physical activity policy through 3 levels: 1) Formal written codes, regulations, or decisions bearing legal authority, such as legislation or zoning; 2) written standards that guide behaviors; and 3) unwritten social norms (1). We suggest that written organizational policies in particular may help codify the intended standard or norm. We describe public policies developed at the federal, state, regional, or local levels that are legislative, litigation-oriented, regulatory, or similar in nature and that can affect support for physical activity simultaneously in multiple worksites in a jurisdiction. Additionally, we identify written standards or policies that can be developed, customized, tailored, and implemented at individual workplaces to increase physical activity.

## Public Policies That Can Affect Physical Activity to, From, and at the Worksite

**Supporting workplace wellness through tax credits.** Wellness tax credits offset the expense of a wellness initiative and can be particularly helpful for encouraging small worksites to offer them, which can indirectly support physical activity as part of a broader initiative. For example, employers in Massachusetts were eligible for an annual wellness tax credit of up to $10,000, or up to 25% of the cost of implementing an employee wellness initiative (2).

**Workplace wellness grants.** Workplace wellness grants are offered by state and local governments specifically designed for small companies. These grants support development of a workplace wellness initiative.

**Workplace wellness incentives.** The Americans with Disabilities Act and the Genetic Information Nondiscrimination Act are relevant legislation for which Equal Employment Opportunity Commission regulations guide worksite health promotion programming and provide incentives to promote employee engagement in wellness initiatives. State legislation can provide tax incentives for comprehensive worksite wellness initiatives, support small businesses with grants to link to community resources to provide wellness support, and amplify or restrict employers’ flexibility with benefit design.

**Employer-offered subsidies for public transportation and active commuting.** Employer subsidies for public transportation can facilitate the use of public transportation and increase physical activity among employees (3). State and local jurisdictions can supplement these subsidies through their own commuter benefit programs. Employers can also promote and support connected, equitable transportation infrastructure and community design that facilitate active modes of transport (rolling, bicycling, and walking) to and from work.

**Complete Streets.** Complete Streets policies integrate all modes of transportation and account for the needs of all road users in an equitable way through the planning, design, operation, and maintenance of transportation networks (4). A Complete Streets approach requires that users of all ages, incomes, and abilities are considered in all roadway construction, repair, and even routine maintenance (such as paving and painting) and reconstruction after roadway disturbance (such as utility work). This is relevant because an increasing number of companies are choosing worksite locations that allow people to walk, bicycle, or use public transit. Walkable neighborhoods attract a more educated workforce with graduate and professional degrees, and the per-capita Gross Domestic Product (GDP) is higher in walkable neighborhoods (5).

## Written Policies That Can Be Implemented at Individual Worksites 

Policies that employers can adopt to influence physical activity at the worksite include short activity breaks, paid time to exercise, flex time for physical activity, and physical activity programs implemented as policies.

**Short activity break policy.** To assist employees in decreasing sedentary behavior, employers can implement policies that support opportunities for employees to break up sedentary behavior with standing or moving. Short breaks in sedentary behavior result in short- and long-term improvements in outcomes such as increased physical activity and improved blood triglycerides and glucose (6). The second edition of Physical Activity Guidelines for Americans emphasizes moving more and sitting less (7). Worksite policies could ensure all employees are afforded the opportunity to take activity breaks in support of more movement.

**Paid time to exercise. **Allowing employees to be physically active while on the clock can increase physical activity and employee interest by addressing the “lack of time” barrier many identify. Combined with other types of intervention strategies such as a gym membership, allowing time for exercise can have synergistic effects on physical activity outcomes (8). Although guidance about the ideal duration and frequency of physical activity breaks is limited, it appears that even a 10-to-15-minute break each workday can result in decreased sedentary behavior, reduced stress, improved mood, increased strength, and improved health (9).

**Stretching at the beginning of shifts.** Encouraging attendance at stretch breaks, especially among manual laborers, might decrease the likelihood of musculoskeletal injury. Stretching appears to predominately reduce discomfort or pain and increase range of motion. However, when these breaks are combined with safety “huddles” (ie, method of communication used for daily task and safety planning), other outcomes, such as improved communication and camaraderie, can occur.

**Flex-time for physical activity. **Worksites offering flex-time for physical activity allow employees to “flex” or shift their work schedules within a certain timeframe while maintaining their expected number of work hours. Employees with flex-time, and even those with a perception of having it, are more likely to be physically active. Flex-time appears to be a popular policy with workers and involves little cost for employers to implement.

**Booster break policy.** Employers can implement a policy that ensures employees are given one 10-to 15-minute movement break every workday. These “booster” breaks consist of employees meeting in an open area and performing some type of physical activity for about 15 minutes, together, often led by a coworker.

**Walking meetings.** Employers may consider policies that allow employees to have walking meetings as part of their workday. Such policies may increase employees’ moderate to vigorous physical activity. Walking meetings can be particularly useful when employees are brainstorming ideas or need to be creative.

## Policy as an Essential Component of Comprehensive Physical Activity Initiatives at the Worksite

Policy may be used to create a worksite culture where being physically active is the norm. Policies are most effective when they are supplemented with information, programs, benefit design, and environmental strategies. Supports for policies include educational posters or emails; programs such as exercise classes, team challenges, or a walking club; and benefit design strategies such as discounted or subsidized gym memberships for employees. Environmental strategies could include treadmill desks, central staircases, point-of-decision stair prompts, suggested walking routes in or around an organization’s building, and onsite exercise facilities. Policy alone may be insufficient to prompt the cultural shifts that must occur to achieve physical activity as the default behavior. However, policies have more potential than physical activity programs alone to affect employees’ physical activity (10).

## Strategies for Policy Implementation

Implementation determines the success of policies, and several pragmatic strategies can bolster implementation of worksite physical activity policies. Policy considerations should include how employees will be informed about the policy, what media and materials to use, and where to post the policy in the worksite. Detailed information about the policy needs to be included, such as where the policy is applicable (eg, employees must remain within one mile of the worksite), when the policy is applicable (eg, not during overtime), and when the policy goes into effect. Enforcement includes assigning accountability and consequences for noncompliance. It is useful for a point of contact to be included in communications to whom questions may be directed.

## Future Research

The paucity of research on physical activity policies at the worksite is inconsistent with the significant need for increased physical activity and the often opportune nature of the worksite setting. Employers may need guidance in making the transition from physical activity programming to developing, implementing, and evaluating policies.

Future research could improve our knowledge regarding what physical activity policies are most effective for different sectors, populations, and job types, and it should address health disparities. Considering the impact of physical activity policies, in addition to combination with other strategies (eg, program, environment), it is worthwhile to implement comprehensive intervention approaches.

We suggest that policies at the individual employer level, as well as public policies developed by federal, regional, state, or local jurisdictions, can have an impact on physical activity at the worksite. Implementing written policies may increase physical activity.
